# A first attempt at determining the antibody-specific pattern of *Platynosomum fastosum* crude antigen and identification of immunoreactive proteins for immunodiagnosis of feline platynosomiasis

**DOI:** 10.14202/vetworld.2022.2029-2038

**Published:** 2022-08-23

**Authors:** Babi Kyi Soe, Poom Adisakwattana, Onrapak Reamtong, Panat Anuracpreeda, Woraporn Sukhumavasi

**Affiliations:** 1 The International Graduate Program of Veterinary Science and Technology, Faculty of Veterinary Science, Chulalongkorn University, Bangkok, Thailand; 2 Department of Helminthology, Faculty of Tropical Medicine, Mahidol University, Bangkok, Thailand; 3 Department of Molecular Tropical Medicine and Genetics, Mahidol University, Bangkok, Thailand; 4 Parasitology Research Laboratory, Institute of Molecular Biosciences, Mahidol University, Nakhon Pathom, Thailand; 5 Parasitology Unit, Department of Pathology, Feline Infectious Disease and Health for Excellence Research Unit, Animal Vector-Borne Disease Research Unit, Microbial Food Safety and Antimicrobial Resistance Research Unit, Faculty of Veterinary Science, Chulalongkorn University, Bangkok, Thailand

**Keywords:** candidate antigens, crude worm extracts, immunoblotting, liquid chromatography-tandem mass spectrometry, *Platynosomum fastosum*

## Abstract

**Background and Aim:**

Feline platynosomiasis, also known as lizard poisoning, is a feline hepatic disease caused by the parasitic trematode *Platynosomum fastosum*. Since this helminth resides in biliary ducts and gallbladder, the heavy infection can lead to failure of the hepatobiliary system and can be associated with cholangiocarcinoma. The primary diagnostic tool currently used is conventional fecal microscopy. However, low sensitivity of detection could occur in the case of light infection or biliary obstruction. This study aimed to determine the antibody-specific pattern of *P. fastosum* crude antigen and to identify immunoreactive proteins to develop the immunodiagnostic techniques.

**Materials and Methods:**

We investigated potential antigens specific to *P. fastosum* infection using western blotting. Forty-six samples of cat serum, including 16 *P. fastosum*-infected sera, eight healthy control sera, and 22 sera infected with other endoparasites were used. The sensitivity, specificity, positive predictive value, and negative predictive value of each band were calculated. Immunoreactive bands with high diagnostic values were further analyzed using liquid chromatography-tandem mass spectrometry (LC-MS/MS) to identify the protein components.

**Results:**

Using immunoblotting, three proteins of 72 kDa, 53 kDa, and 13 kDa were found to be immunogenic. LC-MS/MS identified these proteins as a 70 kDa heat shock protein, a hypothetical protein (CRM22_002083) (adenosine triphosphate synthase subunit beta), and histone H2B, respectively.

**Conclusion:**

This study is the first to reveal three proteins that could be candidates for developing diagnostic tools for feline platynosomiasis.

## Introduction

*Platynosomum fastosum*, syn. *Platynosomum illiciens*, and *Platynosomum concinnum* are a cat liver fluke residing in the hepatobiliary system, and prolonged infection can make cats succumb to bile duct cancer [1]. In contrast, feline platynosomiasis, or lizard poisoning, seems to have been neglected as most of the data about its prevalence comes from postmortem findings. A worldwide prevalence of between 15% and 85% has been reported, and the parasite is especially frequent in areas favoring the development of intermediate hosts [2]. Its prevalence between 2019 and 2020 was estimated to be 8.9% in Bangkok and its vicinity, a value achieved by performing three replicates of each sample to increase the sensitivity of parasite egg detection (manuscript in preparation). As cats are predators, they can become infected by hunting lizards, geckos, and isopods carrying infective metacercaria. The snail *Subulina octona* serves as the first intermediate host. Terrestrial lizards and isopods are the second intermediate host, and harbor infective stage metacercaria [3]. Although the disease is mostly subclinical, clinical signs occasionally appear in the case of chronic and/or heavy infection [4]. Histopathological lesions, including cholangitis, cholangiohepatitis, cholecystitis, periductal fibrosis, biliary cirrhosis, and cholangiocarcinoma have been found to be associated with *P. fastosum* infection [1]. Treatment with praziquantel is generally recommended, but its efficacy is still unclear, and the accurate dosage is still unknown [5, 6].

Although the conventional microscopic examination is the primary diagnostic tool used to detect parasite eggs in the feces, the sensitivity of detection by this method is low in cases of light infection and possibly intermittent egg shedding [2]. A conventional fecal examination can only detect the shedding of *P. fastosum* eggs when they are mature and productive, so false negative results can occur if the fecal examination is performed during the prepatent period. In addition, no *P. fastosum* eggs can be detected in cat fecal samples in cases of complete biliary obstruction. Given the aforementioned limitations, serological tests are the method of choice for the detection of exposure to the parasite, especially during active infection, even in the non-productive stage. The reliability of these tests is not clear since common antigens have been reported from different parasitic helminths [7]. The investigation of immunodominant antigens from parasite products could be beneficial in overcoming this issue. For the development of serodiagnosis tests, parasite antigens derived from crude worms, excretory, secretory, and parasite tegument extracts have been shown to be highly immunogenic, and potentially beneficial as a tool for early serodiagnosis [8]. Until now, pre-mortem diagnosis of *P. fastosum* infection relied on conventional fecal microscopic techniques. There have been no comprehensive reports investigating antigenic components and characterizing the potential antigens of *P. fastosum* for developing immunodiagnostics.

In this study, we investigated potential antigens specific to *P. fastosum* infection, using western blot analysis of *P. fastosum* crude antigens against infected cat sera. The diagnostic power of each immunoreactive band or combination of bands was calculated and compared. The protein components of the three immunoreactive bands with the highest sensitivity and specificity were further investigated using liquid chromatography-tandem mass spectrometry (LC-MS/MS).

## Materials and Methods

### Ethical approval

All procedures, including the collection of parasites from the necropsied cats and serum samples, were approved by the Institutional Animal Care and Use Committee (IACUC No. 2031011) and Biosafety Committee (IBC No. 2031054), Faculty of Veterinary Science, Chulalongkorn University, Thailand.

### Study period and location

The study was conducted from August 2018 to June 2021. The parasitological diagnosis of *P. fastosum* by coprological examination was conducted at Parasitology Unit, Faculty of Veterinary Science, Chulalongkorn University. *P. fastosum* adult worm samples were collected from carcasses at Pathology Unit, Faculty of Veterinary Science, Chulalongkorn University. *P. fastosum*-positive and non-*P. fastosum* positive and negative control serum samples were collected from the Small Animal Teaching Hospital, Faculty of Veterinary Science, Chulalongkorn University. Carmine staining, adult worm protein extraction and immunoreactive protein identification were performed at Department of Helminthology, Faculty of Tropical Medicine, Mahidol University.

### Sample collection and identification

Adult *P. fastosum* worms were collected from the liver, bile duct, and gallbladder during necropsy of the carcasses of three infected cats that died from different causes. Physiological saline (0.85% NaCl) was used to thoroughly rinse the dissected liver and gallbladder tissue samples to harvest the parasites. The worm samples were quickly washed with distilled water and prepared for crude worm antigens (CWAs).

To confirm the morphology of *P. fastosum*, conventional and ultrastructural morphology were conducted using the same batch of parasites (manuscript in revision). For parasite identification, the flukes were fixed in 5% formalin and stained overnight with Semichon’s acetic carmine (Carmine, Sigma-Aldrich, Inc., St. Louis, USA), then dehydration was performed using a graded series of ethanol (30%, 50%, 70%, 95%, and 100%) for 10 min each. The specimen was cleared in xylene for 5 min and mounted using a permanent mounting medium (Permount, Thermo Fisher Scientific Inc., Waltham, USA). Subsequently, the specimens were identified according to their morphology, as described by Pinto *et al*. [3].

For molecular taxonomic identification, the DNA extracted from the *P. fastosum* worm samples and fecal egg samples were sequenced and compared to data deposited in GenBank as part of a molecular study (manuscript in preparation). Briefly, the partial ITS1-5.8S region of *P. fastosum* was amplified using a polymerase chain reaction (PCR), and the nucleotide sequences were analyzed using the NCBI BLAST program (https://blast.ncbi.nlm.nih.gov/Blast.cgi). Using PCR, we detected targeted amplicons with the expected size specific to *P. fastosum* from all *P. fastosum* egg-positive samples. The DNA sequence was confirmed as *P. fastosum* using the reference sequence deposited in GenBank (KU987672-KU987674), as described by Nguyen *et al*. [9].

### Preparation of CWAs

The CWAs were prepared according to a protocol previously described by Adisakwattana *et al*. [10]. The parasite specimen was homogenized with lysis buffer containing 0.01 M phosphate-buffered saline (PBS, pH 7.2), 10 mM Tris-HCl, 150 mM NaCl, 0.5% Triton X-100, 10 mM EDTA, and 1 mM PMSF (P-7626, Sigma-Aldrich, Inc.) using a glass tissue homogenizer. The mixture was then sonicated at 20% amplitude for 5 min in an ice bath with a 15 s pulse on and 15 s pulse off, using an ultrasonic processor, the Vibra cell™ VC-505 (Sonics & Materials Inc., Newtown, USA). Subsequently, the suspension was centrifuged at 5000 g for 20 min at 4°C to remove insoluble materials. Finally, the supernatant was collected and measured for protein concentration using the Bradford protein assay [11] with Bio-Rad protein assay reagent kits (Bio-Rad Laboratories Inc., Hercules, USA). Bovine serum albumin was used as a standard.

### Serum samples

A total of 46 serum samples were used in this study (Table-1). *P. fastosum*-positive sera and sera from animals infected with other helminths were obtained from cats whose infection status was screened by coprological examination using conventional microscopy, as previously described [12], and using conventional PCR in another study by our group using a previously published protocol [9]. At least three replicates of each sample were used for conventional microscopy. For the eight healthy control sera, the inclusion criteria for the recruitment of cats were: clinically healthy with no *P. fastosum* egg in feces at the blood collection time, normal liver biochemical profiles, history of regular deworming, and exclusively indoor status. To calculate the specificity, sensitivity, and cross-reactivity, 22 serum samples from cats infected with other common parasites containing nematodes (*Ancylostoma* spp., *Toxocara* spp., *Trichuris* spp., *Strongyloides* spp.), cestodes (*Dipylidium caninum*, *Taenia taeniaeformis*), and protozoa (*Cystoisospora* spp.) were also collected. To produce significant results, individual serum samples were used for immunoblotting.

**Table-1:** Number of serum samples and diagnosis methods.

Species infected	No. of sera	Types of sample	Diagnostic methods
Trematode			
*Platynosomum fastosum*	16	Feces	PBS-ethyl acetate centrifugal sedimentation
		Feces	Conventional PCR
Nematodes			
*Ancylostoma* spp.	5	Feces	PBS-ethyl acetate centrifugal sedimentation
*Toxocara* spp.	5	Feces	PBS-ethyl acetate centrifugal sedimentation
*Trichuris* spp.	2	Feces	PBS-ethyl acetate centrifugal sedimentation
*Strongyloides* spp.	2	Feces	PBS-ethyl acetate centrifugal sedimentation
Cestodes			
*Dipylidium caninum*	2	Feces	PBS-ethyl acetate centrifugal sedimentation
*Taenia taeniaeformis*	2	Feces	PBS-ethyl acetate centrifugal sedimentation
Protozoa			
*Cystoisospora* spp.	4	Feces	PBS-ethyl acetate centrifugal sedimentation
Control			
Healthy	8	Feces	PBS-ethyl acetate centrifugal sedimentation
		Blood	Liver biochemical profiles Tumor markers (CEA and AFP)
Total	46		

PCR=Polymerase chain reaction, CEA=Carcinoembryonic antigen, AFP=Alpha-fetoprotein, PBS=Phosphate buffered saline

### Sodium dodecyl-sulfate polyacrylamide gel electrophoresis (SDS-PAGE) and immunoblotting

SDS-PAGE of CWAs was performed using the Hoefer™ Mighty Small™ II Mini Vertical Electrophoresis System (Thermo Fisher Scientific Inc., Waltham, USA). Aliquots of 15 mg of protein sample were run on SDS polyacrylamide gels consisting of a 15% separating and a 4% stacking gel, as previously described [13]. Pre-stained standard protein markers (Bio-Rad) covering the range of 10–170 kDa were used for calibration, and the gel was run at a constant current of 40 mA for approximately 2 h at 25°C until the dye front reached the bottom of the gel plate. After electrophoresis, the protein patterns were visualized using Coomassie Brilliant Blue G staining overnight or silver staining (Sigma-Aldrich, Inc.).

Immunoblotting was performed as described by Adisakwattana *et al*. [10] and Towbin *et al*. [14]. Briefly, the proteins from the gel were transferred to nitrocellulose membrane (Bio-Rad) using a semi-dry blotting apparatus, HorizeBlot (Atto Co., Tokyo, Japan), for 100 min at 140 mA. The whole blotting process was done in the transfer buffer containing 25 mM Tris, 192 mM glycine, 0.2% SDS, and 20% methanol. After the transfer process, the nitrocellulose membrane was air-dried and cut into 0.3 cm strips. Nonspecific binding was blocked with blocking solution [5% skimmed milk in 1× PBST (PBS with 0.05% Tween^®^ 20, Biotium Inc., Fremont, USA, pH 7.4)] for 1 h. Subsequently, the membrane strips were quickly washed with 1× PBST and incubated with *P. fastosum*-infected cat serum samples as the primary antibody, diluted 1:100 with 5% skimmed milk in 1× PBST, overnight. Each strip was thoroughly washed four times with 1× PBST for 5 min, then reacted with horseradish peroxidase-conjugated goat anti-feline Immunoglobulin G (IgG) (Southern Biotech, Birmingham, USA) at a dilution of 1:1000 with 5% skimmed milk in 1× PBST for 1 h. The strips were washed with 1× PBST 4 times for 5 min each. Finally, the color reaction was developed by adding 6 mg of 3,3′-diaminobenzidine substrate (Sigma-Aldrich, Inc.). Distilled water was used to stop the enzymatic reaction. All steps were performed at 25°C using a rocking platform shaker. To evaluate the specificity and sensitivity of these antigenic proteins, sera from healthy control cats and cats infected with other parasites (*Ancylostoma* spp., *Toxocara* spp., *Cystoisospora* spp., *D. caninum*, *T. taeniaeformis*, *Trichuris* spp., and *Strongyloides* spp.) were also tested. The immunoblot strips were scanned using a Bio-Rad Image Scanner (Bio-Rad).

### LC-MS/MS analysis

The immunogenic bands were cut and subjected to gel tryptic digestion using an in-gel digestion method as described by Chienwichai *et al*. [15]. Briefly, the gel was destained in 50% acetonitrile until it became colorless, and then all solution was removed. Then 10 mM dithiothreitol was added to reduce the disulfide bonds. The gel pieces were then alkylated in 10 mM iodoacetamide in the dark for 1 h, after which the solution was discarded. The gel fragments were then added to acetonitrile and stored at 25°C for 5 min. The proteins in the gel plug were immersed in 10 ng/mL trypsin and incubated in 30% acetonitrile at 37°C overnight. Subsequently, the peptide products were extracted from the gel fragments using 50% acetonitrile and 0.1% formic acid and kept at −80°C until use. The tryptic peptide samples were then analyzed for amino acid sequences using a tandem mass spectrometer coupled with a nano-liquid chromatography system (Thermo Fisher Scientific Inc.). Data from the mass spectrometry analysis were searched against the platyhelminth NCBI database with the MASCOT search engine (http://www.matrixscience.com/) (Matrix Science, London, UK) to identify the amino acid sequence. The search parameters accounted for the trypsin digestion and monoisotopic mass and allowed a maximum of one missed cleavage. The peptide and fragment mass tolerance were set as 0.8 Da and 0.8 Da, respectively. Variable modifications were set to carbamidomethylation of cysteine and oxidation of methionine. Proteins with a significant match score (p < 0.05) were reported. Finally, the protein ID scores, peptide matches, and percentage of sequence coverage were evaluated.

### Statistical analysis

The sensitivity, specificity, positive predictive value, and negative predictive value of each immunogenic band were calculated using 2 × 2 matrix table as described by Anuracpreeda *et al*. [8]. For further clinical and field practice, the cumulative probability of the diagnostic potential between immunogenic proteins was also tested.

## Results

### Immunopatterns specific to platynosomiasis in cats

**Figure-1:**
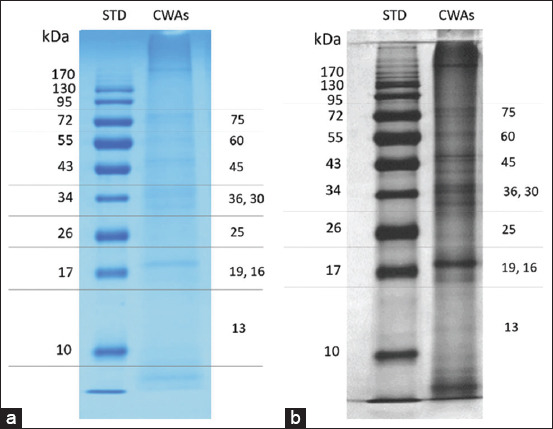
SDS-PAGE protein patterns of CWAs of *Platynosomum fastosum*. CWAs (15 μg) were separated using 15% SDS-PAGE. Numbers on the right margin show the different ranges of protein patterns using (a) Coomassie staining; (b) silver staining. STD=Standard molecular weight marker, CWAs=Crude worm antigens, SDS-PAGE=Sodium dodecyl-sulfate polyacrylamide gel electrophoresis.

**Figure-2:**
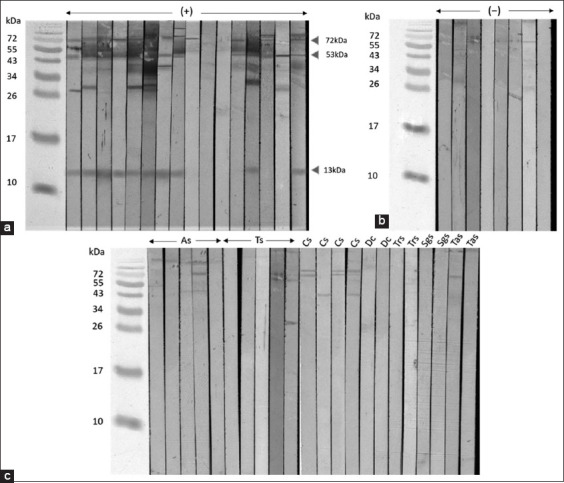
Western blot analyses of *Platynosomum fastosum* crude worm antigens using *P. fastosum*-infected cat sera, healthy control sera, and sera infected with other endoparasites as probes. (a) The bands at 72 kDa, 53 kDa, and 13 kDa (arrowheads) were detected using sera from *P. fastosum*-infected cats (n = 16) with a high percentage of reactivity. (b) The weak band at 72 kDa was detected in 50% (4 out of 8 cats) using sera from healthy control cats (n = 8). (c) Western blot analyses using sera from cats infected with other endoparasites (n = 22). As=*Ancylostoma* spp., Ts, *Toxocara* spp., Cs=*Cystoisospora* spp., Dc=*Dipylidium caninum*, Trs=*Trichuris* spp., Sgs=*Strongyloides* spp., Tas=*Taenia taeniaeformis*.

On the SDS-PAGE gels, *P. fastosum* crude worm extracts showed protein bands with molecular weights ranging from 120 to 13 kDa. Distinct bands appeared at 75 kDa, 60 kDa, 45 kDa, 36 kDa, 30 kDa, 25 kDa, 19 kDa, 16 kDa, and 13 kDa (Figures-1a and b). Immunoblotting was performed to determine the specific immunogenic pattern of *P. fastosum*-infected cat serum. Seven major proteins were found in the sera of *P. fastosum*-infected cats. Their molecular weights were 72 kDa, 60 kDa, 53 kDa, 43 kDa, 37k Da, 30 kDa, and 13 kDa. The percentage of immunoreactivity of each antigenic component against the sera of *P. fastosum*-infected cats, healthy control cats, and cats infected with other parasites are shown in Table-2. Of these seven major bands, those at 72 kDa, 53 kDa, and 13 kDa were found to be immunogenic proteins since their reactivity percentages were 81.25% (13/16), 81.25% (13/16), and 62.5% (10/16), respectively. Immunoblotting results for all serum samples tested are shown in Figures-2a-c.

**Table-2:** Immunoreactivity of components antigenic to *P. fastosum*-infected sera, healthy control sera, and sera from animals infected with other parasites.

	72 kDa (%)	60 kDa (%)	53 kDa (%)	43 kDa (%)	37 kDa (%)	30 kDa (%)	13 kDa (%)
*P. fastosum-* infected cases	81.25 (13/16)	31.25 (5/16)	81.25 (13/16)	18.75 (3/16)	25 (4/16)	31.25 (5/16)	62.5 (10/16)
Healthy control	50.00 (4/8)	−	−	−	−	−	−
Other parasites- infected cases	18.18 (4/22)	13.63 (3/22)	−	9.09 (2/22)	−	−	−

−=Nothing appeared.* P. fastosum*=*Platynosomum fastosum*

### Analysis of sensitivity, specificity, and predictive values

The individual and cumulative sensitivity, specificity, positive predictive values, and negative predictive values of these three immunogenic bands are shown in Table-3. Of these three bands, those at 53 kDa and 13 kDa showed 100% specificity, whereas that at 72 kDa had 78.94% specificity. A sensitivity of 84.21% was obtained for the 72 kDa and 53 kDa proteins, whereas the 13 kDa had a sensitivity of 72.72%. The cumulative sensitivity and specificity of these three immunogenic bands were calculated using two options, either one protein or another and both proteins, as referred as either/or and both/and. With the either/or option, the sensitivity percentage was increased and the specificity was slightly decreased. However, when we analyzed either the 53 kDa or 13 kDa proteins, both sensitivity and specificity became higher, at 88.88% and 100%, respectively. When both proteins were analyzed together, the specificity was increased to 100% in all cases, but the sensitivity was decreased.

**Table-3:** Cumulative sensitivity, specificity, positive predictive value, and negative predictive value of potential antigenic bands.

	Sensitivity (%)	Specificity (%)	Predictive values (%)	

			Positive	Negative
72 kDa	84.21	78.94	66.66	90.90
53 kDa	84.21	100	100	90.90
13 kDa	72.72	100	100	83.33
72 or 53 kDa	94.11	78.94	66.66	96.77
72 or 13 kDa	88.88	78.94	66.66	93.75
53 or 13 kDa	**88.88**	**100**	100	93.75
72 and 53 kDa	**84.21**	**100**	100	90.90
72 and 13 kDa	69.56	100	100	81.08
53 and 13 kDa	69.56	100	100	81.08
72, 53 and 13 kDa	69.56	100	100	81.08

High percentage of sensitivities and specificities (in bold) occurred in two conditions: using either 53 kDa or 13 kDa and using both 72 kDa and 53 kDa proteins

### Mass spectrometry analysis

After the immunoblot analysis, potential candidate proteins were investigated. After the bands of interest were excised and digested with trypsin, the resulting peptide digests were identified using a nano LC-MS/MS. According to the MASCOT results, a 70 kDa heat shock protein (HSP70) from *Paragonimus westermani*, a hypothetical protein (CRM22_002083) (adenosine triphosphate (ATP) synthase subunit beta) of *Opisthorchis felineus*, and histone H2B proteins of *Trichobilharzia regenti* had the highest MS scores, and several peptides matched with the 72 kDa, 53 kDa, and 13 kDa bands. The protein accession numbers in the database, protein scores, theoretical molecular weight, and peptide matches are shown in Table-4.

**Table-4:** Identification of candidate proteins from crude worm extracts of *Platynosomum fastosum* using a nano LC-MS/MS.

Protein (MW)	Protein (helminths)	Accession no.	Theoretical MW	Protein score	No. of matched peptides
72 kDa	70 kDa Heat shock protein (*Paragonimus westermani*)	KAA3675093.1	70	658	34
53 kDa	Hypothetical protein CRM22_002083 (ATP synthase subunit b) (*Opisthorchis felineus*)	TGZ72442.1	55	236	15
13 kDa	Histone H2B (*Trichobilharzia regenti*)	VDQ05944.1	13	82	4

LC-MS/MS=Liquid chromatography-tandem mass spectrometry, MW=Molecular weight

## Discussion

In this study, we aimed to determine the antibody-specific pattern of *P. fastosum* crude antigens, and to identify immunoreactive proteins to develop immunodiagnostic applications. Many *P. fastosum*-infected cats have been diagnosed at the necropsy table. This observation could imply that the disease is still overlooked, possibly because it is mostly asymptomatic [2]. Since neoplastic changes in the biliary epithelium have been reported, *P. fastosum* is considered to be an etiologic agent for cholangiocarcinoma [1]. Despite its veterinary importance, the genomic dataset of the cat liver fluke *P. fastosum* is still poorly understood. Interest in using a serodiagnosis approach has increased, since this approach could detect the presence of the parasite even when it is in the prepatent period [16]. The presence of PCR inhibitors in fecal egg samples can affect PCR reaction [17].

Furthermore, PCR approach has not been used in on-site clinics, as expensive equipment and trained personnel are required [18]. Given these problems, the development of early serodiagnosis tools is important. Until now, there has been no information about *P. fastosum* antigens that might be useful in developing serodiagnosis tools. In this study, CWAs of *P. fastosum* were investigated. Three proteins were considered to be immunogenic and further analyzed using liquid chromatography-tandem mass spectrometry.

Three proteins, with weights of 72 kDa, 53 kDa, and 13 kDa, showed a high percentage of immunoreactivity, of 81.25%, 81.25%, and 62.5%, respectively. Although the 72 kDa protein showed 81.25% immunoreactivity against *P. fastosum*-infected sera, this protein was also found to react with 50% (4/8) of healthy control cat sera. This reactivity could be due to previous *P. fastosum* infection of healthy control cats, resulting in the formation of an immune complex with remaining circulatory antibodies. Although our recruitment criteria were stringent, only a single fecal examination was performed. Antibodies are produced following exposure of the host to a pathogen, and this production is affected by the host’s immunologic status, so the development of detection methods that can distinguish the current active infection from the previous infection is important [19]. To overcome this issue, antibody-based serodiagnosis to detect antigens against a specific parasite [20], or antibody subclass determination, should be further pursued since the detection of the earliest immune responses could be beneficial [21]. The relationship between parasite exposure and the development of parasite-specific antibody responses has previously been investigated, in situations such as *Schistosoma mansoni* infection [22] and *Opisthorchis viverrini* infection [23], in which IgG4 was found to be involved in the development of protective immunity and the intensity of infection.

The cumulative sensitivity and specificity of the three immunogenic proteins were analyzed to evaluate the potential of serodiagnosis for clinical usage. The 72 kDa and 53 kDa proteins had a sensitivity of 84.21%, whereas the 13 kDa-sized protein had a sensitivity of 72.72%. However, sensitivity was increased when the either/or condition of the three proteins was chosen. Thus, the option of either/or should be used to identify *P. fastosum*-infected cats with high sensitivity using the three proteins. In contrast, 100% specificity was shown for the 53 kDa and 13 kDa proteins, whereas the 72 kDa protein was detected with a sensitivity of 78.94%. When we tested a combination of the 72 kDa and 53 kDa proteins, the specificity reached 100% with unchanged sensitivity. For the three immunogenic proteins, 100% specificity occurred in two conditions: using either the 53 kDa or the 13 kDa protein, and using both 72 kDa and 53 kDa proteins, to rule out non-infected cases. Therefore, we suggest that either the 53 kDa or the 13 kDa protein might be a good candidate for the definitive diagnosis of feline platynosomiasis with a sensitivity of 88.88% and a specificity of 100%, respectively. Sera derived from cats infected with *O. felineus* or *Clonorchis sinensis*, could be included in further investigations to address whether cross-immunoreactivity with parasite families other than *P. fastosum* could occur.

Some studies have investigated the diagnostic potential of antigens from parasite crude worm extracts [8, 20, 24–27]. For instance, 37 kDa and 28 kDa antigens were detected in CWAs of *C. sinensis*, in which recombinant cathepsin L protease (35kDa) was revealed as a reliable diagnostic antigen with a sensitivity of 96% and a specificity of 96.2% [24]. The diagnostic value of recombinant HSP70 has been analyzed out of six immunodominant proteins (120 kDa, 110 kDa, 95 kDa, 80 kDa, 70 kDa, and 65 kDa) in schistosomiasis [25, 26]. Moreover, 100 kDa, 70 kDa, 43 kDa, and 37 kDa proteins were identified as major immunoreactive bands for *O. viverrini* infection in humans, a finding which led to the evaluation of the diagnostic potential of recombinant cathepsin B1 (44 kDa) [27, 28]. Compared to these helminths, *P. fastosum* had specific immunodominant antigens of 72 kDa, 53 kDa, and 13 kDa.

A lack of data is a barrier to the further development of recombinant technology, as few sequences of *P. fastosum* have been described to date. At present, mass spectrometry is an indispensable tool for the identification of proteins of interest [29]. The candidate antigens revealed by immunoblotting were therefore characterized using mass spectrometry. Although it may not be possible to compare the sequences produced using mass spectrometry within the same species, we were able to check with other closely related helminths. From the LC-MS/MS analysis, an HSP70, a hypothetical protein (CRM22_002083) (ATP synthase subunit beta), and histone H2B proteins were determined to be the most significant immunogenic proteins.

Heat shock proteins are known to be molecular chaperones and are classified into different families according to their molecular weights. They primarily involve protein synthesis, modifying processes, and several immune responses [30]. Since HSPs can elicit pro- and anti-inflammatory cytokines, these proteins also play pivotal roles in innate immune responses. Due to the multi-host life cycle of parasites, they have to overcome the dissimilar condition of growth by the parasite throughout their biological cycle. Parasite HSPs play an integral part in dealing with hostile environments by mediating environmental stresses and cellular homeostasis [31]. Heat shock proteins have been considered to be major immunogens in parasites, recognized by humoral immune responses [32] and detected by immunohistochemistry studies [33]. As previously described, the fusion of bovine HSPs and DNA vaccines encoded with triose phosphate isomerase (SjCTPI) or the tetraspanin membrane protein (SjC23) of *Schistosoma japonicum* has been shown to reduce fecal miracidial hatching, which in turn reduced in worm burden and the transmission of schistosomiasis [34]. Heat shock protein 70 has also been considered as a diagnostic candidate for helminth infections such as schistosomiasis in a murine model and in humans, as well as trichinellosis in rabbits, pigs, and rodents [32, 35, 36]. Recombinant major egg antigens (HSPs) of *P. westermani* have been shown to be a target antigen for the serodiagnosis of paragonimiasis [37]. We, therefore, suggest that HSP70 proteins could be major constituents of crude worm extracts of *P. fastosum* and could be useful as candidate antigens for immunodiagnosis.

In this study, the 53 kDa protein appeared to be involved in ATP synthesis as an ATP synthase b subunit. Mitochondrial ATP synthase is a complex enzyme with multiple subunits and is essential for oxidative phosphorylation under physiological conditions. Adenosine triphosphate synthase plays a critical role in a number of mitochondrial functions, such as the formation of reactive oxygen species, Ca^2+^ homeostasis, and tumor growth. Desensitization of the permeability transition pore by ATP synthase has been found to promote apoptosis in the primary phase of cancer development. Since abnormal regulation of ATP synthase subunits has occurred in different cancer cell lines, this enzyme has been considered a novel target for cancer treatment [38]. ATPase plays a key role in nematode metabolism and is located mainly in the mitochondria, intracellular fluid, and microsomes. ATPase may, therefore, be involved in parasite behavior and responses during infections [39]. Adenosine triphosphate synthase is one of the most abundant proteins in *Schistosoma mekongi* eggs [40] and adult *O. viverrini* flukes [41]. In mosquitos infected with malaria, the ATP synthase subunit gene was disrupted by ablating the protein that converts ADP to ATP, resulting in the reduction of asexual parasite growth [42]. Until now, few studies have investigated this enzyme’s involvement in helminth infection. Since parasite mitochondria play a pivotal role in parasite survival and adaptation to the host environment [43], their potential roles in diagnosing *P. fastosum* infection require further investigation.

Thirteen H2B isoforms have been shown to be involved in posttranslational modifications such as acetylation, methylation, and phosphorylation [44]. Histones are also involved in immune responses by producing antimicrobial proteins from their N-terminus end [45]. In the complex parasite life cycle, histone proteins play pivotal roles in epigenetic mechanisms, especially parasite development and response to environmental cues, through posttranslational modifications mediated by chromatin-linked proteins [46]. Recently, histones and histone modifying enzymes were shown to be novel drug targets for protection against parasitic infection [47]. Despite their unclear role, the upregulation of histones could produce chromatin modifications, resulting in misfolding or mispackaging chromatin, which may lead to DNA damage [48]. Therefore, the targeting of parasite chromatin proteins becomes of considerable interest. In the previous studies, histone has been shown to be secreted copiously by *S. japonicum* and *O. viverrini* [41, 49]. *Plasmodium falciparum* histones have recently been demonstrated to be a causal agent involved in the pathogenesis of cerebral malaria [50]. It was, therefore, interesting to see this protein identified as immunogenic in the current study. However, the diagnostic potential of histones in helminth infection remains unclear. In this study, we identified histone proteins as neglected novel antigens, and thus the possibility of recombinant histone proteins for the diagnosis of feline platynosomiasis should be pursued further.

Recombinant antigens are reliable and practical to use in serodiagnosis, since cross-reactivity can be reduced and reproducible results produced [51]. Recombinant protein production would, therefore, be a valuable avenue of future research. However, recombinant techniques are dependent on existing databases, and there are very limited sequences available for *P. fastosum*. Further recombinant protein studies of these three immunogenic proteins would therefore be challenging. To solve this problem, a genome database must be established for *P. fastosum*. We investigated three immunogenic proteins and established their diagnostic potential for further clinical application.

## Conclusion

This study is the first attempt to identify specific antigens that could be used for the serodiagnosis of *P. fastosum* infection. Since most of the *P. fastosum*-infected cats were diagnosed at postmortem examination, serodiagnosis would be helpful to determine the presence of infection regardless of prepatent infection, light infection, or biliary obstruction. We investigated the diagnostic potential of three immunogenic proteins. If an early diagnosis can be made before biliary tract hyperplasia, treatment with specific anthelminthic drugs and proper care can lead to successful outcomes. Although a genome database for *P. fastosum* is not yet available, the potential utility of the three immunogenic proteins for serodiagnosis was discussed. In veterinary practice, large sample sizes should be used in further field studies. Furthermore, further studies of immunoblotting using different antigens and the immunolocalization of the antigens identified should be performed to extend our knowledge.

## Authors’ Contributions

WS and PAd: Conceptualization, methodology. PAd: Supervision of protein-related laboratory work. BKS: Collection of the samples, laboratory work, data analysis and drafting of the manuscript. OR: Supervision of mass spectrometry analysis. WS, PAd, OR, and PA: Manuscript revision. WS: Supervision of ethical form preparation, parasite identification and isolation, communication with and approach cat owners for blood collection. All authors have read and approved the final manuscript.

## References

[1] Andrade R.L, Dantas A.F, Pimentel L.A, Galiza G.J, Carvalho F.K, Costa V.M, Riet-Correa F (2012). *Platynosomum fastosum*-induced cholangiocarcinomas in cats. Vet. Parasitol.

[2] Basu A.K, Charles R.A (2014). A review of the cat liver fluke *Platynosomum fastosum* Kossack, 1910 (*Trematoda*:Dicrocoeliidae). Vet. Parasitol.

[3] Pinto H.A, Mati V.L, de Melo A.L (2014). New insights into the life cycle of *Platynosomum* (*Trematoda*:Dicrocoeliidae). Parasitol. Res.

[4] Ramos R.A, Lima V.F, Monteiro M.F, de Andrade Santana M, Lepold R, Faustino M.A, Rinaldi L, Cringoli G, Alves L.C (2016). New insights into diagnosis of *Platynosomum fastosum* (*Trematoda*:Dicrocoeliidae) in cats. Parasitol. Res.

[5] Lathroum C.N, Shell L, Neuville K, Ketzis J.K (2018). Efficacy of praziquantel in the treatment of *Platynosomum fastosum* in cats with natural infections. Vet. Sci.

[6] Shell L, Ketzis J, Hall R, Rawlins G, du Plessis W (2015). Praziquantel treatment for *Platynosomum* species infection of a domestic cat on St Kitts, West Indies. J. Feline Med. Surg. Open Rep.

[7] Ngoc D.P, Arimatsu Y, Kaewkes S, Sripa B (2015). Characterization of immunogenic *Clonorchis sinensis* protein fractions by gel filtration chromatography. Asian Pac. J. Trop. Dis.

[8] Anuracpreeda P, Chawengkirttikul R, Sobhon P (2016). Antigenic profile, isolation and characterization of whole body extract of *Paramphistomum gracile*. Parasit. Immunol.

[9] Nguyen H.M, Van Hoang H, Ho L.T (2017). *Platynosomum fastosum* (*Trematoda*:Dicrocoeliidae) from cats in Vietnam:Morphological redescription and molecular phylogenetics. Korean J. Parasitol.

[10] Adisakwattana P, Viyanant V, Chaicumpa W, Vichasri-Grams S, Hofmann A, Korge G, Sobhon P, Grams R (2007). Comparative molecular analysis of two asparaginyl endopeptidases and encoding genes from *Fasciola gigantica*. Mol. Biochem. Parasitol.

[11] Bradford N (1976). A rapid and sensitive method for the quantitation microgram quantities of a protein isolated from red cell membranes. Anal. Biochem.

[12] Jitsamai W, Khrutkham N, Hunprasit V, Chandrashekar R, Bowman D, Sukhumavasi W (2021). Prevalence of endoparasitic and viral infections in client-owned cats in metropolitan Bangkok, Thailand, and the risk factors associated with feline hookworm infections. Vet. Parasitol. Reg. Stud. Rep.

[13] Laemmli U.K (1970). Cleavage of structural proteins during the assembly of the head of bacteriophage T4. Nature.

[14] Towbin H, Staehelin T, Gordon J (1979). Electrophoretic transfer of proteins from polyacrylamide gels to nitrocellulose sheets:Procedure and some applications. Proc. Natl. Acad. Sci. U. S. A.

[15] Chienwichai P, Ampawong S, Adisakwattana P, Thiangtrongjit T, Limpanont Y, Chusongsang P, Chusonsang Y, Reamtong O (2020). Effect of praziquantel on *Schistosoma mekongi* proteome and phosphoproteome. Pathogens.

[16] Arifin N, Hanafiah K.M, Ahmad H, Noordin R (2019). Serodiagnosis and early detection of *Strongyloides stercoralis* infection. J. Microbiol. Immunol. Infect.

[17] Laude A, Valot S, Desoubeaux G, Argy N, Nourrisson C, Pomares C, Machouart M, Le Govic Y, Dalle F, Botterel F, Bourgeois N (2016). Is real-time PCR-based diagnosis similar in performance to routine parasitological examination for the identification of *Giardia intestinalis*, *Cryptosporidium parvum*/*Cryptosporidium hominis* and *Entamoeba histolytica* from stool samples?Evaluation of a new commercial multiplex PCR assay and literature review. Clin. Microbiol. Infect.

[18] Chan K, Wong P.Y, Yu P, Hardick J, Wong K.Y, Wilson S.A (2016). A rapid and low-cost PCR thermal cycler for infectious disease diagnostics. PLoS One.

[19] Murray P.R, Rosenthal K.S, Pfaller M.A (2020). Medical Microbiology E-Book.

[20] Anuracpreeda P, Chawengkirttikul R, Sobhon P (2016). Immunodiagnosis of *Fasciola gigantica* infection using monoclonal antibody-based sandwich ELISA and immunochromatographic assay for detection of circulating cathepsin L1 protease. PLoS One.

[21] Saavedra-Langer R, Marapara J, Valle-Campos A, Durand S, Vásquez-Chasnamote M.E, Silva H, Pinedo-Cancino V (2018). IgG subclass responses to excreted-secreted antigens of *Plasmodium falciparum* in a low-transmission malaria area of the Peruvian Amazon. Malar. J.

[22] Egesa M, Lubyayi L, Jones F.M, van Diepen A, Chalmers I.W, Tukahebwa E.M (2018). Antibody responses to *Schistosoma mansoni* schistosomula antigens. Parasit. Immunol.

[23] Rodpai R, Luvira V, Sadaow L, Sukeepaisarnjaroen W, Kitkhuandee A, Paonariang K, Sanpool O, Ittiprasert W, Mann V.H, Intapan P.M, Brindley P.J (2022). Rapid assessment of *Opisthorchis viverrini* IgG antibody in serum:A potential diagnostic biomarker to predict risk of cholangiocarcinoma in regions endemic for opisthorchiasis. Int. J. Infect. Dis.

[24] Nagano I, Pei F, Wu Z, Wu J, Cui H, Boonmars T, Takahashi Y (2004). Molecular expression of a cysteine proteinase of *Clonorchis sinensis* and its application to an enzyme-linked immunosorbent assay for immunodiagnosis of clonorchiasis. Clin. Diag. Lab. Immunol.

[25] Kanamura H.Y, Hancock K, Rodrigues V, Damian R.T (2002). *Schistosoma mansoni* heat shock protein 70 elicits an early humoral immune response in *S. mansoni* infected baboons. Mem. Inst. Oswaldo Cruz.

[26] Sulahian A, Garin Y.J.F, Izri A, Verret C, Delaunay P, van Gool T, Derouin F (2005). Development and evaluation of a Western blot kit for diagnosis of schistosomiasis. Clin. Diag. Lab. Immunol.

[27] Choi M.H, Ryu J.S, Lee M, Li S, Chung B.S, Chai J.Y, Hong S.T (2003). Specific and common antigens of *Clonorchis sinensis* and *Opisthorchis viverrini* (*Opisthorchidae*, *Trematoda*). Korean J. Parasitol.

[28] Sripa J, Brindley P.J, Sripa B, Loukas A, Kaewkes S, Laha T (2012). Evaluation of liver fluke recombinant cathepsin B-1 protease as a serodiagnostic antigen for human opisthorchiasis. Parasitol. Int.

[29] Zhang F.K, Hu R.S, Elsheikha H.M, Sheng Z.A, Zhang W.Y, Zheng W.B, He J.J (2019). Global serum proteomic changes in water buffaloes infected with *Fasciola gigantica*. Parasit. Vectors.

[30] Abaza S (2014). Heat shock proteins and parasitic diseases:Part 1:Helminths. J. Egypt. Parasitol. United.

[31] Chen H.Y, Cheng Y.S, Shih H.H (2017). Heat shock proteins:Role, functions and structure in parasitic helminths. Heat Shock Proteins in Veterinary Medicine and Sciences.

[32] Sotillo J, Valero L, Del Pino M.M.S, Fried B, Esteban J, Marcilla A, Toledo R (2008). Identification of antigenic proteins from *Echinostoma caproni* (*Trematoda*) recognized by mouse immunoglobulins M, A and G using an immunoproteomic approach. Parasit. Immunol.

[33] Higón M, Monteagudo C, Fried B, Esteban J, Toledo R, Marcilla A (2008). Molecular cloning and characterization of *Echinostoma caproni* heat shock protein-70 and differential expression in the parasite derived from low-and high-compatible hosts. Parasitology.

[34] Da'dara A.A, Li Y.S, Xiong T, Zhou J, Williams G.M, McManus D.P (2008). DNA-based vaccines protect against zoonotic schistosomiasis in water buffalo. Vaccine.

[35] Liu F, Cui S.J, Hu W, Feng Z, Wang Z.Q, Han Z.G (2009). Excretory/secretory proteome of the adult developmental stage of human blood fluke, *Schistosoma japonicum*. Mol. Cell. Proteomics.

[36] Wang S, Zhu X, Yang Y, Yang J, Gu Y, Wei J, Cui S (2009). Molecular cloning and characterization of heat shock protein 70 from *Trichinella spiralis*. Acta Trop.

[37] Lee J.S, Lee J, Kim S.H, Yong T.S (2007). Molecular cloning and characterization of a major egg antigen in *Paragonimus westermani* and its use in ELISA for the immunodiagnosis of paragonimiasis. Parasitol. Res.

[38] Galber C, Acosta M.J, Minervini G, Giorgio V (2020). The role of mitochondrial ATP synthase in cancer. Biol. Chem.

[39] Dhaka M.S, Srivastava S, Bhattacharya S.M (2016). Role and significance of various ATPases of nematode parasites. In:Regulation of Ca^2+^-ATPases, V-ATPases and F-ATPases.

[40] Thiangtrongjit T, Adisakwattana P, Limpanont Y, Dekumyoy P, Nuamtanong S, Chusongsang P, Chusongsang Y, Reamtong O (2018). Proteomic and immunomic analysis of *Schistosoma mekongi* egg proteins. Exp. Parasitol.

[41] Mulvenna J, Sripa B, Brindley P.J, Gorman J, Jones M.K, Colgrave M.L, Loukas A (2010). The secreted and surface proteomes of the adult stage of the carcinogenic human liver fluke *Opisthorchis viverrini*. Proteomics.

[42] Sturm A, Mollard V, Cozijnsen A, Goodman C.D, McFadden G.I (2015). Mitochondrial ATP synthase is dispensable in blood-stage *Plasmodium berghei* rodent malaria but essential in the mosquito phase. Proc. Natl. Acad. Sci. U. S. A.

[43] Hikosaka K, Komatsuya K, Suzuki S, Kita K (2015). Mitochondria of malaria parasites as a drug target. An Overview of Tropical Diseases.

[44] Peterson C.L, Laniel M.A (2004). Histones and histone modifications. Curr. Biol.

[45] Li C, Song L, Zhao J, Zhu L, Zou H, Zhang H, Cai Z (2007). Preliminary study on a potential antibacterial peptide derived from histone H2A in hemocytes of scallop *Chlamys farreri*. Fish Shellfish Immunol.

[46] Vilcinskas A (2016). The role of epigenetics in host-parasite coevolution:Lessons from the model host insects *Galleria mellonella* and *Tribolium castaneum*. Zoology (*Jena*).

[47] Nawaz M, Malik I, Hameed M, Kuthu Z.H, Zhou J (2020). Modifications of histones in parasites as drug targets. Vet. Parasitol.

[48] Molden R.C, Bhanu N.V, LeRoy G, Arnaudo A.M, Garcia B.A (2015). Multi-faceted quantitative proteomics analysis of histone H2B isoforms and their modifications. Epigenetics Chromatin.

[49] Liu M, Ju C, Du X.F, Shen H.M, Wang J.P, Li J, Hu W (2015). Proteomic analysis on cercariae and schistosomula in reference to potential proteases involved in host invasion of *Schistosoma japonicum* larvae. J. Proteome. Res.

[50] Moxon C.A, Alhamdi Y, Storm J, Toh J.M, McGuinness D, Ko J.Y, Toh C.H (2020). Parasite histones are toxic to brain endothelium and link blood barrier breakdown and thrombosis in cerebral malaria. Blood Adv.

[51] Basu K, Green E.M, Cheng Y, Craik C.S (2019). Why recombinant antibodies benefits and applications. Curr. Opin. Biotechnol.

